# Host-pathogen systems biology: logical modelling of hepatocyte growth factor and *Helicobacter pylori *induced c-Met signal transduction

**DOI:** 10.1186/1752-0509-2-4

**Published:** 2008-01-14

**Authors:** Raimo Franke, Melanie Müller, Nicole Wundrack, Ernst-Dieter Gilles, Steffen Klamt, Thilo Kähne, Michael Naumann

**Affiliations:** 1 Institute of Experimental Internal Medicine, Otto von Guericke University, Leipziger Str. 44, 39120 Magdeburg, Germany; 2 Max Planck Institute for Dynamics of Complex Technical Systems, Sandtorstrasse 1, 39106 Magdeburg, Germany

## Abstract

**Background:**

The hepatocyte growth factor (HGF) stimulates mitogenesis, motogenesis, and morphogenesis in a wide range of tissues, including epithelial cells, on binding to the receptor tyrosine kinase c-Met. Abnormal c-Met signalling contributes to tumour genesis, in particular to the development of invasive and metastatic phenotypes. The human microbial pathogen *Helicobacter pylori *can induce chronic gastritis, peptic ulceration and more rarely, gastric adenocarcinoma. The *H. pylori *effector protein cytotoxin associated gene A (CagA), which is translocated via a type IV secretion system (T4SS) into epithelial cells, intracellularly modulates the c-Met receptor and promotes cellular processes leading to cell scattering, which could contribute to the invasiveness of tumour cells. Using a logical modelling framework, the presented work aims at analysing the c-Met signal transduction network and how it is interfered by *H. pylori *infection, which might be of importance for tumour development.

**Results:**

A logical model of HGF and *H. pylori *induced c-Met signal transduction is presented in this work. The formalism of logical interaction hypergraphs (LIH) was used to construct the network model. The molecular interactions included in the model were all assembled manually based on a careful meta-analysis of published experimental results. Our model reveals the differences and commonalities of the response of the network upon HGF and *H. pylori *induced c-Met signalling. As another important result, using the formalism of minimal intervention sets, phospholipase Cγ1 (PLCγ1) was identified as knockout target for repressing the activation of the extracellular signal regulated kinase 1/2 (ERK1/2), a signalling molecule directly linked to cell scattering in *H. pylori *infected cells. The model predicted only an effect on ERK1/2 for the *H. pylori *stimulus, but not for HGF treatment. This result could be confirmed experimentally in MDCK cells using a specific pharmacological inhibitor against PLCγ1. The *in silico *predictions for the knockout of two other network components were also verified experimentally.

**Conclusion:**

This work represents one of the first approaches in the direction of host-pathogen systems biology aiming at deciphering signalling changes brought about by pathogenic bacteria. The suitability of our network model is demonstrated by an *in silico *prediction of a relevant target against pathogen infection.

## Background

*H. pylori *is a highly successful micro-aerophilic spiral-shaped bacterium that has colonized the gastric epithelium of half of the human population [1,2]. *H. pylori *is a major risk factor for peptic ulcer disease, gastric cancer and gastric mucosa-associated lymphoid tissue (MALT) lymphoma [3]. It was the first bacterial pathogen to be classified as a class I carcinogen by the WHO. Gastric cancer remains the second deadliest cancer worldwide, which makes *H. pylori *infection, also in light of growing bacterial resistances to antibiotics, a significant global health problem [4].

*H. pylori *has evolved elaborate mechanisms to manipulate host cells during infection. Following colonization of the gastric epithelial apical surface and adhesion, various *H. pylori *virulence factors interfere with signalling pathways in gastric epithelial cells. The presence of a pathogenicity island (cag PAI) in *H. pylori *is strongly associated with the development of gastric diseases. The cag PAI encodes a T4SS that mediates translocation of bacterial virulence factors into the host cell [5]. The three major *H. pylori *virulence factors involved in bacterial-epithelial interactions that are associated with an increased risk of severe gastritis, gastric atrophy and/or gastric cancer, are the cag pathogenicity island (cag PAI), the vacuolating cytotoxin A (VacA), and the blood group antigen-binding adhesionA2 (BabA2), which binds Lewis B on gastric epithelial cells [3]. CagA, one of the main virulence factors of *H. pylori*, also encoded in the PAI, is translocated via the T4SS into the host cell cytoplasm, where it modulates cellular functions. Attachment of CagA-positive *H. pylori *induces cell scattering in human gastric epithelial cells [6]. Cell scattering comprises cell spreading and elongation, and the cells become motile. Therefore, cell scattering is one readout for the motogenic response of *H. pylori *infected cells. Recent studies have shown that CagA intracellularly modulates the receptor tyrosine kinase c-Met [6]. Binding of the natural ligand HGF to c-Met stimulates mitogenesis, motogenesis, and morphogenesis in epithelial cells [7]. Abnormal c-Met signalling has been strongly related to tumour genesis, in particular to the development of invasive and metastatic phenotypes [8]. Numerous experiments indicate a particular role of HGF and the proto-oncogene c-Met in tumour invasive growth [6]. It has been shown that c-Met signalling induced by *H. pylori *leads to the activation of ERK1/2 in AGS cells [6]. ERK1/2 activity promotes cell scattering in a transcription independent manner. It has also been shown that activation of ERK1/2 is critical for the induction of cell scattering in *H. pylori*-infected epithelial cells [6], which could contribute to the invasiveness of tumour cells. Therefore, blocking the activation of ERK1/2 represents a promising intervention goal to prevent *H. pylori *induced signalling changes, which could play a role for cancer metastasis.

The induction of cell scattering by *H. pylori *in epithelial cells, is an example how human microbial pathogens modulate signal transduction in the cell by translocated bacterial proteins. The presented work aims at translating these complex interactions into a logical network model.

Signalling networks have not yet been modelled at a scale comparable to metabolic and regulatory networks. More than 500 members of the protein kinase superfamily of enzymes alone are encoded in the human genome, which allows for an enormous complexity of signalling. The wealth of data generated, describing signalling networks in molecular detail at a rapidly increasing rate makes the reconstruction of such large networks a difficult task [9,10]. The most often used formalism to model cellular networks is kinetic analysis, which has been applied to signalling networks of smaller size [11] or for modelling single pathways [12]. A large scale reconstruction of signalling networks relying on kinetic data has not yet appeared due to the lack of available kinetic data for the interactions in the network. The data obtained by recent Genomics and Proteomics high throughput technologies are often only qualitative or semi-quantitative. Therefore, qualitative (i.e. parameter-free) modelling seems to be the only feasible approach at the moment to represent and analyse large-scale signalling networks in a computer. A functional analysis of the network structure already enables to address important issues, such as detection of network-wide interdependencies, identification of intervention strategies and qualitative predictions on the effect of perturbations.

In our view it would be invalid to try to construct a complete network model of *H. pylori *infection due to the limited number of detailed information about the cellular processes triggered by this pathogen. Thus, in contrast to a *H. pylori *infection model, we explicitly only considered the signal transduction events that directly arise from c-Met-receptor-mediated signalling, which becomes modulated by the *H. pylori *virulence factor CagA.

In order to construct a qualitative network model of c-Met activation by HGF and *H. pylori *we used here a methodology introduced previously [13,14] relying on logical interaction hypergraphs (LIH). Our model reveals the differences and commonalities of the response of the network upon HGF and *H. pylori *induced c-Met signalling. Another goal of this study was to use the logical model to generate *in silico *predictions and to verify these experimentally. As one case study demonstrating the predictive capabilities of our model, we determine suitable interventions that prevent an activation of ERK1/2, because of the above mentioned decisive role of ERK1/2 for cell scattering and tumour invasive growth.

## Results and Discussion

### Logical modelling of signal transduction networks

**Figure 1:**
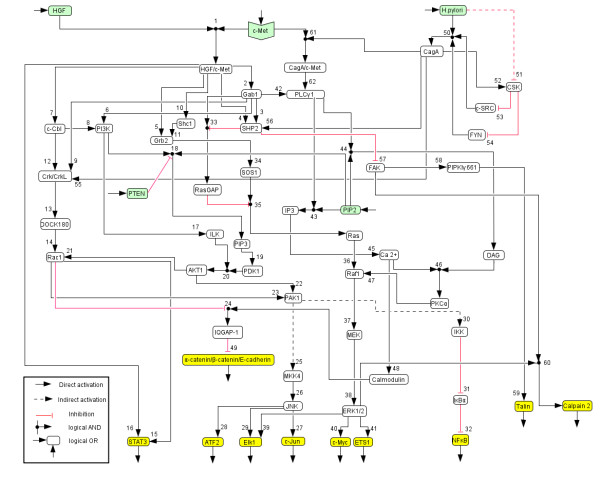
**Logical model of HGF and *H. pylori *induced c-Met signalling**. Hyperarcs are numbered corresponding to the network description in the text.

For the reconstruction and qualitative analysis of the signal flow network we employ a logical modelling framework (Boolean networks represented as *logical interaction hypergraphs*) as introduced previously [13,14]. Boolean network modelling for biological systems has so far mainly been applied to the analysis of medium-scale regulatory networks [15-18]. In contrast to regulatory networks (whose behaviour is mainly determined by their regulatory feedback loops), signalling networks are much stronger structured in input, intermediate and output layers and the input signals usually govern the response of the network. For this characteristic network topology we introduced *logical interaction hypergraphs *(LIHs) [13] as a special representation of Boolean networks, which is well suited to formalize, visualize and analyse logical models of signal transduction networks. As in all Boolean networks, nodes in the network represent species (e.g. kinases, adaptor molecules or transcription factors) each having an associated logical state (in the binary case as used herein only "on" (1) or "off" (0)) determining whether the species is active (or present) or not. Signalling events are encoded as Boolean operations on the network nodes. For example, protein kinase C alpha (PKCα) can be activated (gets "on", i.e. value 1) if the level of calcium AND of diacylglycerol (DAG) is high, i.e. calcium and DAG must be "on" to activate PKCα (see connection 46 in Figure 1). Usually, a node can be activated by more than one signalling event, which are then OR-connected, e.g. the MAPKKK Raf1 becomes active if either Ras OR PKCα is active (Figure 1).

In general, in LIHs we make only use of the Boolean operators AND (·), OR (+), and NOT (!), which are sufficient to represent any logical relationship. A signalling event in a LIH is an AND connection of nodes (negation of node values using the NOT operator are allowed) describing one opportunity how the target species of this connection can be activated. Hence, for the first example described above we would write

DAG AND Ca^2+ ^→ PKCα or shorter DAG ·Ca^2+ ^→ PKCα

In a graphical representation of the network (see PKCα node in Figure 1), such an AND connection is displayed as a *hyperarc*. In contrast to arcs in graphs, a hyperarc (in hypergraphs) may have several start or end nodes. Clearly, in some cases, only one species is involved in activating another as in the case

Ras → Raf1.

In these special cases, the hyperarc is a simple arc as occurring in graphs; we will nevertheless refer to it as a hyperarc. As already pointed out, a species may be activated via several distinct signalling events (hyperarcs), i.e. all these signalling events are OR-connected. This is illustrated by Raf1, which can be activated via

Ras → Raf1 OR PKCα → Raf1.

Accordingly, all the hyperarcs pointing into a species are OR connected. In this way we can easily interpret Figure 1, which displays graphically the interactions given in Table 1.

**Table 1:** Hyperarcs of the logical model

-> c-Met
-> HGF
-> *H. pylori*
-> PIP2
-> PTEN
ATF2 ->
α-catenin/β-catenin/E-cadherin ->
Calpain ->
c-Myc ->
Elk1 ->
ETS1 ->
NF-κB ->
Talin ->
c-Jun ->
STAT3 ->

01.) HGF**·**c-Met -> HGF/c-Met
02.) HGF/c-Met -> Gab1
03.) Gab1 -> SHP2
04.) HGF/c-Met -> SHP2
05.) HGF/c-Met -> Grb2
06.) Gab1 -> PI3K
07.) HGF/c-Met -> c-Cbl
08.) c-Cbl -> PI3K
09.) Gab1 -> Crk/CrkL
10.) HGF/c-Met -> Shc1
11.) Shc1 -> Grb2
12.) c-Cbl -> Crk/CrkL
13.) Crk/CrkL -> DOCK180
14.) DOCK180 -> Rac1
15.) RAC1 -> STAT3
16.) HGF/c-Met -> STAT3
17.) PI3K -> ILK
18.) !PTEN**·**PI3K**·**PIP2 -> PIP3
19.) PIP3 -> PDK1
20.) PDK1**·**ILK -> AKT1
21.) AKT1 -> RAC1
22.) AKT1 -> PAK1
23.) Rac1 -> PAK1
24.) Rac1**·**Calmodulin -> IQGAP-1
25.) PAK1 -> MKK4
26.) MKK4 -> JNK
27.) JNK -> c-JUN
28.) JNK -> ELK1
29.) JNK -> ATF2
30.) PAK1 -> IKK
31.) !IKK -> IκBα
32.) !IκBα -> NF-κB
33.) !SHP2**·**Gab1 -> RasGAP
34.) Grb2 -> SOS1
35.) !RasGAP**·**SOS1 -> Ras
36.) Ras -> Raf1
37.) Raf1 -> MEK
38.) MEK -> ERK1/2
39.) ERK1/2 -> ELK1
40.) ERK1/2 -> c-Myc
41.) ERK1/2 -> ETS
42.) Gab1 -> PLCγ1
43.) PLCγ1**·**PIP2 -> IP3
44.) PLCγ1**·**PIP2 -> DAG
45.) IP3 -> Ca^2+^
46.) DAG**·**Ca^2+ ^-> PKCα
47.) PKCα -> Raf1
48.) Ca^2+ ^-> Calmodulin
49.) !IQGAP-1 -> α-catenin/β-catenin/E-cadherin
50.) c-SRC**·**FYN**·***H. pylori *-> CagA
51.) !*H. pylori *-> CSK
52.) CagA -> CSK
53.) !CSK -> c-SRC
54.) !CSK -> FYN
55.) CagA -> Crk/CrkL
56.) CagA -> SHP2
57.) !SHP2 -> FAK
58.) FAK -> PIPKIγ661
59.) PIPKIγ661 -> Talin
60.) FAK**·**ERK1/2 -> Calpain 2
61.) CagA**·**c-Met -> CagA/c-Met
62.) CagA/c-Met -> PLCγ1

Exclamation mark denotes a logical NOT and dots within the equation indicate AND operations.

Once we have constructed an LIH, we may start to analyse it. A typical scenario is that we apply a pattern of inputs to the network and we would like to know how the nodes in the network will respond to this stimulation. As explained in [13], by propagating input signals along the logical (hyperarc) connections (which is equivalent to compute the logical steady state resulting from the input stimuli) we obtain the qualitative response of the network. It depends on the functionality of positive or negative feedback loops in the network whether we can resolve a complete and unique logical response of all nodes for a given set of input stimuli (for example, negative feedback loops may prevent the existence of a logical steady state [13]). Feedback loops are usually present in signalling networks, however, one can often identify at least one connection in each loop that becomes active at a later time-scale and does not play a significant role for the early signalling events. Setting these late-event connections inactive, one obtains an acyclic network for which always a unique network response for a given set of inputs can be computed. Using this technique, one can easily perform *in silico *experiments, for example check how knockouts (or knockins) alter the network response.

With the idea of minimal intervention sets (MISs, [13,14]) one may even directly search for those interventions that enforce a desired response (e.g. inactivation of a transcription factor). As described by Klamt et al. [14], MISs can be computed by testing systematically which combinations of knockouts (KOs) and knockins (KIs) fulfil a specified intervention goal. One usually starts with the single KOs and KIs: one clamps the logical value (0 or 1, respectively) of the respective node, computes the resulting logical steady state (as explained above) and verifies whether the intervention goal is achieved. All those KOs and KIs, that were not successful are then combined in pairs which may lead to some MISs of size two. Then all combinations with 3 interventions (that are not a superset of the MISs of size one or two) are tested and so forth. Obviously, especially computing the MISs of higher cardinality becomes a highly combinatorial problem and one usually restricts oneself to the low cardinality MISs. There are also some heuristics that can be used to accelerate the computation [14].

Another advantage of LIHs is, that we can easily derive the (signed and directed) interaction graph underlying the logical model: we only have to split hyperarcs with two or more start nodes (i.e. the AND connections) into simple arcs. Interaction graphs cannot be used to give on/off predictions, however, they provide an appropriate formalism to search for signalling paths and feedback loops. Another useful feature that can be extracted from interaction graphs is the *dependency matrix *as introduced in [13,14] which displays network-wide interdependencies between all pairs of species. For example, a species A is an activator (inhibitor) of another species B, if at least one path leads from A to B and if all those paths are positive (negative). This kind of information can be very useful for predicting effects of perturbations.

The model studied in this work was implemented in and analysed with *CellNetAnalyzer*, a comprehensive toolbox for functional analysis of cellular networks [14].

### Logical network of HGF and *H. pylori *triggered c-Met signal transduction

The logical network of HGF and *H. pylori *induced c-Met signal transduction was constructed as a bottom up approach. We restricted our network model explicitly to the signal transduction events that arise from stimulation or modulation of the c-Met receptor. Other virulence factors of *H. pylori *than CagA that target different receptors of the host epithelial cell are therefore not included in the model. The modeling objective was to construct a logical network model that is capable to predict qualitatively how CagA modulates the c-Met receptor, and to compare HGF versus CagA induced c-Met signal transduction. Only accurate and well-defined interactions were included in the model. The molecular interactions were all assembled manually by extensive use of literature search engines and databases (see Methods). Only data obtained from epithelial cell lines were considered for the model. The data were subjected to a careful meta-analysis; only data that were consistent with the current knowledge and did not interfere with recent publications were taken.

Activation of the c-Met receptor by its endogenous ligand HGF leads to autophosphorylation of specific tyrosine residues in its cytoplasmic domain within the intracellular activation loop (Y1234 and Y1235) resulting in an activation of the intrinsic kinase activity. Subsequently, a multifunctional signal transducer docking site is formed by phosphorylation of Y1349 and Y1356 [19]. This docking site recruits intracellular adapters via Src homology-2 domains (SH2), phosphotyrosine binding domains (PTB) and Met binding domains (MBD).

Recent studies have shown that infection of gastric epithelial cells by the bacterial pathogen *H. pylori *targets the c-Met receptor and provokes some c-Met-related cellular responses [6]. This includes especially the enhancement of cell scattering, which is mainly mediated by the bacterial virulence factor CagA, translocated during infection via a T4SS into gastric epithelial cells.

In the following, the biochemical steps included in the logical model will be described briefly. The numbering corresponds to the hyperarcs in Figure 1 and Table 1.

1. HGF is the natural ligand of c-Met. Upon ligand binding, c-Met undergoes autophosphorylation of specific tyrosine residues within the intracellular region. Phosphorylation of Y1230, Y1234 and Y1235 located within the activation loop of the tyrosine kinase domain activates the intrinsic kinase activity of c-Met [19].

2. Gab1 is subsequently recruited to activated c-Met by direct binding to the tyrosine phosphate residues Y1349 and Y1356 of the receptor [20].

3.-7. The c-Met/Gab1 complex forms a multivalent binding site for a number of downstream molecules, including SHP2, PI3K, Grb2.

3. Fusion of Gab1 with c-Met induces tyrosine phosphorylation and interaction with SHP2 [21].

4. Activated c-Met also interacts with SHP2 independently of Gab1 [22].

5. The adaptor protein Grb2 binds to the Y1356 docking site of c-Met [23].

6. c-Met associates withPI3K via Gab1 [23,24].

7. c-Cbl is a substrate of the activated tyrosine kinase receptor c-Met [25].

8. PI3K can also be activated by c-Cbl [26].

9. Gab1 interacts with Crk and CrkL, two proteins with SH2 and SH3 protein interaction domains. This interaction is mediated via the SH2 domains [27].

10. The c-Met receptor associates with the Shc1 adaptor, via the SH2 domain [28].

11. Grb2 binds to Shc via its SH2-domain [29].

12. Phosphorylated c-Cbl interacts with the SH2 domain of Crk/CrkL [25].

13. Activated Crk/CrkL recruits DOCK180 through its SH3 domain [30].

14. DOCK180 binds and activates Rac1 [31].

15. Rac1 directly binds and phosphorylates the transcription factor STAT3 [32].

16. Activated c-Met directly phosphorylates STAT3 [33].

17. PI3K activates ILK [34].

18. PI3K converts the plasma membranelipid PIP2 to PIP3 [35]. The phosphatase PTEN selectively removes the 3-phosphate of PIP3 to regenerate PIP2, counteracting PI3K activity [36].

19. PDK1 is a key PIP3-binding protein [37].

20. Akt1 is subsequently activated by PDK1 and ILK [34,38].

21. Akt1 phosphorylates a single serine residue of Rac1 [39].

22. Akt1 activates PAK1 [40].

23. Activation of Rac1 leads to the activation of PAK1 [39,41].

24. Rac1 interacts with IQGAP-1, thereby crosslinking actin filaments. Under these conditions IQGAP-1 does not bind to β-catenin and cannot dissociate α-catenin from the cadherin-catenin complex, leading to strong adhesion. When Rac1 is not active, activated Calmodulin allows IQGAP-1 to interact with β-catenin to dissociate α-catenin from the cadherin-catenin complex [42].

25.,26. PAK1 stimulates JNK activity through a MAP kinase regulatory cascade. PAK1 regulates the activity of an unknown MAP kinase kinase kinase, which controls activity of MKK4. We used a dotted hyperarc between PAK1 and MKK4 to indicate an unknown compound that links PAK1 to MKK4 [43].

27. JNK phophorylates the transcription factor c-Jun [44].

28. JNK phosphorylates the transcription factor Elk1 [45].

29. JNK phosphorylates ATF2 on Thr-69 and Thr-71 [46].

30.-32. HGF activates NF-κB through an Akt1 -> PAK1 pathway [40]. PAK1 is required for the activation of NF-κB by activating the IKK complex through an unknown kinase. The hyperarc connecting PAK1 and IKK is therefore also shown as unknown link. IκBα is phophorylated by the IKK complex and undergoes ubiquitin-mediated degradation, allowing nuclear translocation of NF-κB [40].

33. Gab1 interacts with RasGAP SH2 domains, only under conditions when SHP2 is not activated. SHP2 downregulates RasGAP by dephosphorylating RasGAP binding sites on Gab1 [47,48].

34. Grb2 forms a complex with SOS1 and recruits it to the plasma membrane [49].

35. SOS1 is an exchange factor and activator of Ras, which is downregulated by RasGAP [50].

36.-38. Activated Ras activates the Raf1 kinase, which in turn activates the MAP kinase MEK. MEK activation leads to ERK1/2 activation [51,52]. Ras proteins activate at least three families of downstream effector signalling pathways, involving Raf kinases, phosphatidylinositol 3 (PI 3)-kinase, and Ral-specific guanine nucleotide exchange factors (Ral-GEFs) [53]. PI3K is also a downstream component of the adaptor Gab1, as such it is included in our model. We did not include the Ras/PI3K/PIP3 and the Ras/Ral-GEF/Ral pathways, because they are not described in the context of c-Met signaling. For our model, we therefore only considered the Ras-Raf-MEK-ERK pathway.

39. ERK1/2 phosphorylates Elk1 on its C-terminal activation domain [54].

40. ERK1/2 phosphorylates and stabilizes c-Myc [55].

41. ERK1/2 phosphorylates ETS1 [56].

42. Phosphorylated Gab1 recruits PLCγ1 [57].

43.,44. PLCγ1 hydrolyses PIP2 to produce IP3 and DAG [58].

45. IP3 raises the Calcium level by opening Ca^2+^-channels [59].

46. The activation of PKCα is Ca^2+^-dependent. Provided a high Ca^2+^-level, DAG binds to and activates PKCα [59,60].

47. PKCα promotes activation of Raf1 by direct phosphorylation [61,62].

48. Calmodulin is a Ca^2+^-receptor protein and is regulated by the Ca^2+^-level [63].

49. IQGAP-1 is a regulator of E-cadherin mediated cell-cell adhesion. When Rac1 is not active, activated Calmodulin allows IQGAP-1 to interact with β-catenin to dissociate α-catenin from the cadherin-catenin complex. This leads to a reduction of cell-cell adhesions [42,64,65].

50. Attached *H. pylori *translocates CagA via T4SS [66]. Upon membrane localization translocated CagA undergoes subsequent tyrosine phosphorylation by c-Src and Fyn [67]. Thus the node CagA in the logical network corresponds to phosphorylated CagA.

51. Whereas resting host cells are characterized by almost inactive Src kinases due to phosphorylation of their regulatory loops by C-terminal Src kinase (CSK), it is known that *H. pylori *can transiently activate src family kinases by an unknown mechanism, which is figuratively shown as an inactivation of CSK [68].

52.-54. Nascently phosphorylated CagA activates CSK and thereby leads to a subsequent inactivation of c-Src and Fyn [69]. As the activation of CSK via CagA occurs significantly later than the activation of other downstream events in the network, we set the time scale parameter of connection 52 on 2. Experimental data presented by Tsutsumi *et al*. in 2003 [69] provide the basis for setting up two time scale scenarios. In the early events the translocated CagA protein undergoes tyrosine phophorylation by Src family kinases (see step 51). In the later events tyrosine phophorylated CagA binds and activates CSK, which in turn phosphorylates and inactivates Src family kinases. The phosphorylation of CagA has to occur at an earlier timepoint, because CagA-CSK interaction involves the SH2-domain of CSK and is strictly dependent on CagA tyrosine phophorylation [69]. CSK then works as a negative regulator of CagA-Shp2 signalling, because the inactivated Src family kinases lead to a down-regulation of the levels of CagA phophorylation and subsequent diminished CagA-Shp2 complex formation.

In 2002 Mimuro *et al*. published data, that suggested that CagA could interact with Grb2 and thereby activate Ras-Raf-Mek-ERK [70]. This observation was clearly contradicted later by other groups. The groups of Hatakeyama [71] and Naumann [6] were not able to reproduce the published results of Mimuro *et al*.. In light of these results and after careful evaluation of the available data we decided not to include the direct interaction between CagA and Grb2 in our network model.

55. Phosphorylated CagA directly binds to the adaptor proteins Crk and CrkL [72].

56. Phosphorylated CagA binds and activates SHP2 [73].

57. SHP2 inactivates FAK by dephosphorylation and thereby induces/enhances cell scattering [74].

58. FAK phosphorylates PIPKIγ661 [75].

59. Tyrosine phosphorylated PIPKIγ661 associates with Talin [75].

60. FAK physically associates with Calpain2 and spatially couples it to its upstream regulator ERK1/2 [76].

61. CagA intracellularly interacts with phosphorylated c-Met [6].

62. In contrast to HGF activated c-Met, CagA replaces Gab1 and/or Grb2 and leads to PLCγ1 activation [6].

The model was built on the current understanding of the biochemical processes involved in HGF and *H. pylori *induced c-Met signal transduction and iteratively improved by validation with published experimental results. If the proposed model failed to reproduce the experimental results, it was modified accordingly.

In total, the network model contains 54 species and 62 hyperarcs (plus 15 input and output arcs). The set of nodes includes 5 input elements (HGF and *H. pylori *as the events starting the signalling cascade, PTEN as externally regulated signal and PIP2 and c-Met as externally provided constituents that are always present in the cell) and 10 elements in the output layer (among these the seven transcription factors STAT3, ATF2, Elk1, c-Jun, c-Myc, ETS1, NF-κB).

### Network analysis: induction of the signalling cascades by HGF and by *H. pylori*

We first analysed the interaction graph underlying the logical network. Two (negative) feedback loops can be found in the network regulating CagA phosphorylation by src family kinases. These loops, however, are not active during the early events because both feedback loops involve connection 52 (CagA → CSK) having a time-scale parameter of 2. The computation of signalling paths revealed that 86 paths connect the input node HGF with one of the output nodes, whereas *H. pylori *may influence the ouput nodes only via 63 signalling paths (but every output node can still be reached). Below we will discuss the reason for this difference in the number of signalling paths and show what the consequences of this reduced flexibility is.

**Figure 2:**
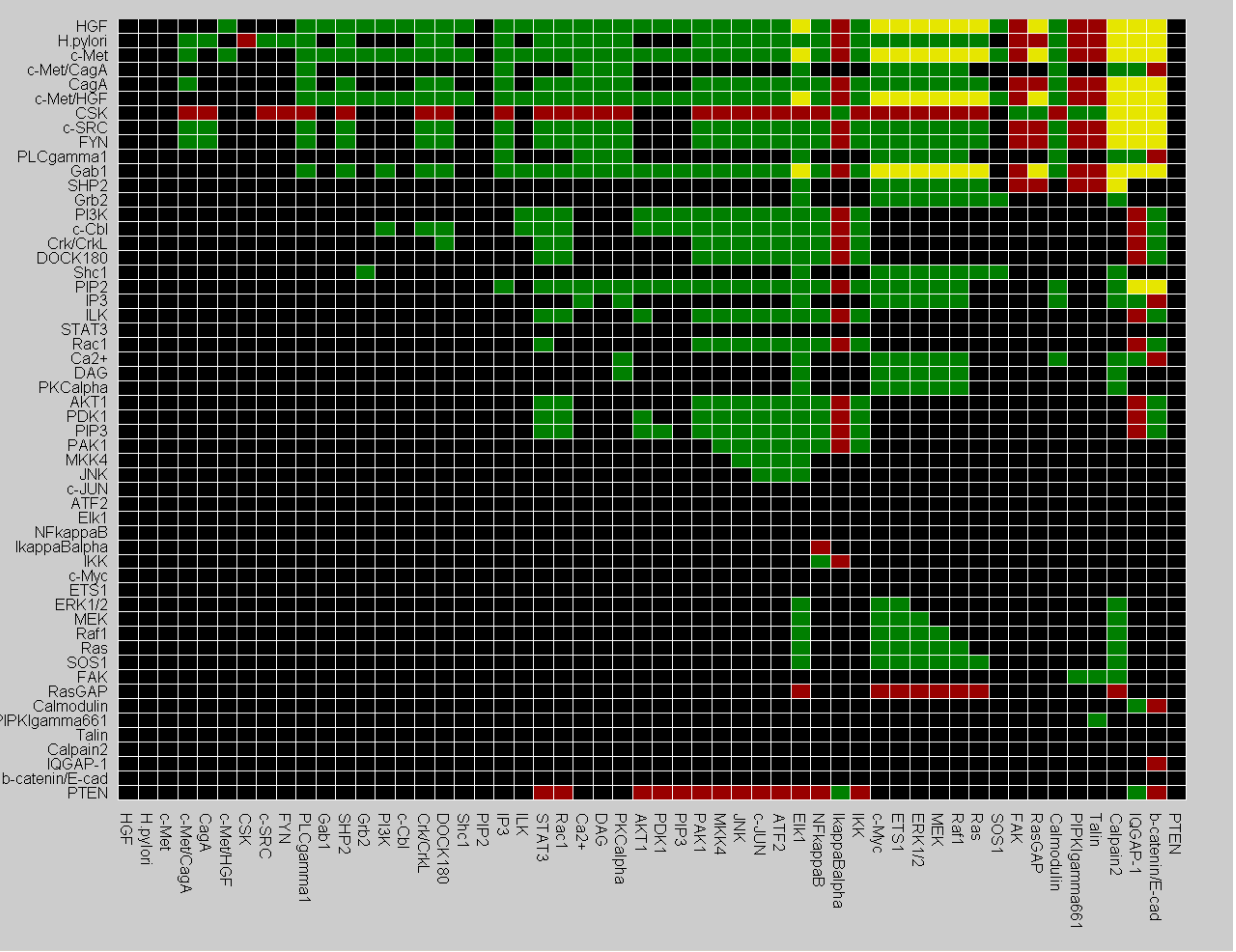
**Dependency matrix**. The dependency matrix displays network-wide interdependencies in the logical model. The colour of matrix element m_ij _defines the type of the impact of element *i *on *j *(green: (strong) activator; red: (strong) inhibitor, yellow: ambivalent factor; black: no effect (see [13])).

The dependency matrix of the interaction graph (computed for time scale 1, Figure 2) displays all functional dependencies between each pair of species. In the following we discuss some of these dependencies. HGF is an activator for the transcription factors STAT3, ATF2, c-Jun and NF-κB, i.e. there are only positive paths from HGF to these nodes which can thus only mediate activating effects. In contrast, HGF is an ambivalent factor for ERK1/2 and its downstream effectors Elk1, c-Myc and ETS1, i.e. there is at least one inhibiting and one activating path emanating from HGF to these species. The reason is that HGF has a positive effect on ERK1/2 via Grb2-Sos1-Ras-Raf1-MEK but does also signal through RasGAP, which has an inhibitory effect on Ras and therefore on the MAP kinase cascade. However, we notice that the activity of RasGAP on the other hand is downregulated by SHP2 so that the above pathway running over Ras is functional. In contrast to HGF, *H. pylori *is an activator for all seven transcription factors because ERK1/2 cannot be activated via Grb2-Sos1-Ras-Raf1-MEK- and therefore only through the positive pathway PKCα-Raf1-MEK-ERK1/2 (the latter is also functional with HGF). Another example for coexisting positive and negative effects is the α-catenin/β-catenin/E-cadherin-complex, for which both *H. pylori *and HGF are ambivalent factors, because they signal through Rac1, which has an inhibitory effect on IQGAP-1, and via calmodulin with a positive effect.

**Figure 3:**
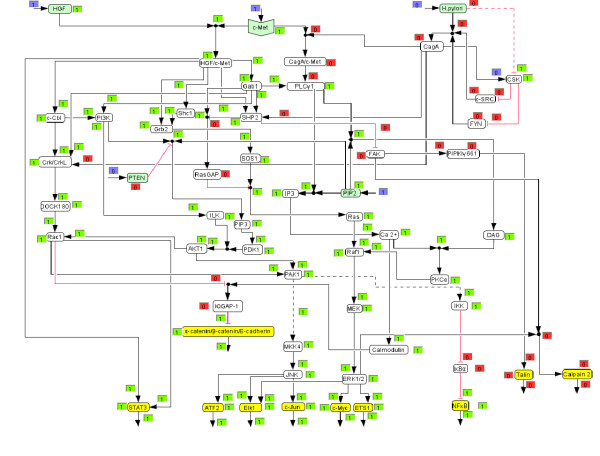
**HGF triggered signalling network (screenshot of *CellNetAnalyzer)***. Each species and each hyperarc has an associated text box displaying a logical value. Blue boxes indicate clamped (fixed) values prior computing the logical steady state (i.e. the network response). Green boxes ("on") indicate an active species or activating signal flow, respectively. Red boxes ("off") indicate inactive (or absent) species or, in the case of hyperarcs, connections along which no activation of the end species of the hyperarc takes place.

**Figure 4:**
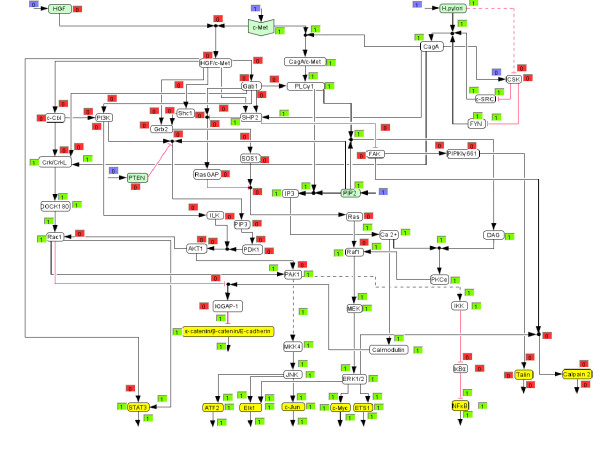
***H. pylori *stimulated network**. See also legend of Figure 3.

We then used the logical model to compute the response of the network (i) when the network is triggered with HGF (Figure 3; *H. pylori *stimulation is off) and (ii) when c-Met is stimulated by *H. pylori *(Figure 4; HGF = 0), both for the early events (connection 52 is off).

Interestingly, the resulting on/off states for the output nodes are identical for both triggers, but the signalling pathways that lead to these differ significantly (Figures 3 and 4). The main cause for these differences lies in the fact that *H. pylori *modulates the c-Met receptor by translocating CagA, which replaces adaptor molecules like Gab1 and Grb2 at the c-Met receptor and leads to PLCγ1 activation [6]. Therefore, signalling events, including the whole PI3K pathway, that originate from the c-Met/Gab1 binding site in the HGF stimulated network are thus not included in the *H. pylori *triggered network. That is also the reason why the number of paths connecting *H. pylori *with the output layer is lower compared to the HGF case.

### Target identification: deactivation of ERK1/2

To evaluate the usefulness and robustness of our model for the generation of *in silico *predictions, deactivation of ERK1/2 in the *H. pylori *triggered network was defined as an intervention goal having biomedical relevance. *In silico *identified targets would provide clues for anti-cancer therapies, as prevention of ERK1/2 activation would have an impact on cell scattering and thus invasiveness of tumour cells [6].

A strong feature of *CellNetAnalyzer *(CNA) is the possibility to compute minimal intervention sets (MISs). This function can be used to predict targets for knockouts (or possibly knockins) that lead to a desired response defined by an intervention goal. Accordingly, ERK1/2 repression (i.e. a logical level of 0) was defined as intervention goal and *H. pylori *input was set to 1 and HGF input set to 0. When searching only for single interventions (i.e. the cardinality of the intervention sets was restricted to 1), CNA calculated 15 minimal intervention sets each proposing one knockout target (*H. pylori*, c-Met, CagA/c-Met, CagA, c-Src, Fyn, PLCγ1, PIP2, IP3, Ca^2+^, DAG, PKCα, ERK1/2, MEK, Raf1). When HGF was the stimulus (*H. pylori *input was set to 0 and HGF input set to 1), CNA finds only 6 knockout targets for ERK1/2 repression (HGF, c-Met, HGF/c-Met, ERK1/2, MEK, Raf1).

By comparing the MISs for the two scenarios ((i) stimulation with *H. pylori *and (ii) stimulation with HGF) we can identify targets, that imply a deactivation of ERK1/2 for scenario (i) but not for (ii). Among those we chose PLCγ1 for further evaluation.

**Figure 5:**
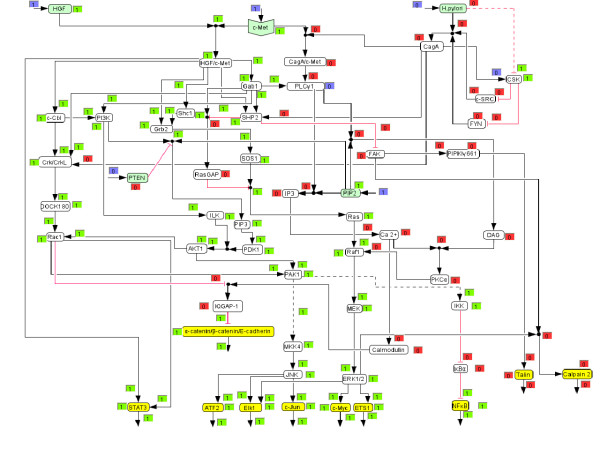
***In silico *knockout of PLCγ1 in the HGF stimulated signalling network**. A knockout of PLCγ1 does not influence the ERK1/2 pathway in the HGF stimulated network, ERK1/2 is still active. Regarding the text box colors see figure 3.

**Figure 6:**
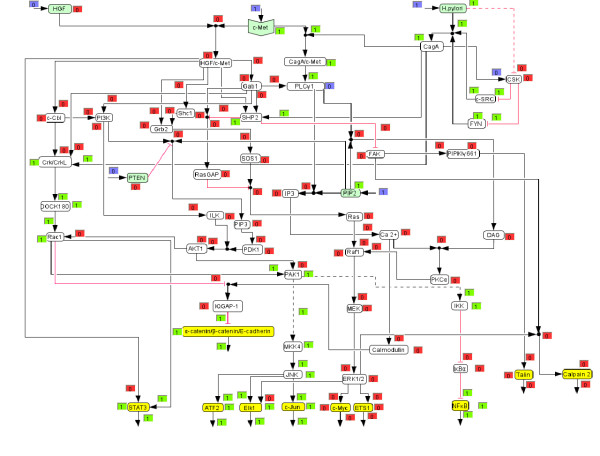
***In silico *knockout of PLCγ1 in the *H. pylori *stimulated network**. In contrast to the HGF activated network scenario, knockout of PLCγ1 in the *H. pylori *stimulated network leads to deactivation of ERK1/2. Regarding the text box colors see figure 3.

Figures 5 and 6 show the computed network response for the *in silico *knockouts of PLCγ1 for *H. pylori *and HGF stimulation, respectively. In agreement with the computed MISs, ERK1/2 is deactivated in the case of *H. pylori *stimulation (Figure 6), but is still activated in the case of HGF activation (Figure 5), demonstrating the different response of the network for the two stimuli. Our model shows that the knockout of PLCγ1 can be bypassed via Grb2 – SOS1 – Ras – Raf1 – MEK in the case of HGF stimulation. Next we wanted to verify this prediction experimentally. Apart from PLCγ1, we also selected PI3K (no qualitative effect on ERK1/2 in both scenarios) and MEK (which is, for both cases, a MIS for deactivating ERK1/2) as two further knockout candidates to be tested in experiments.

### Experimental validation of the predicted results from the model

**Figure 7:**
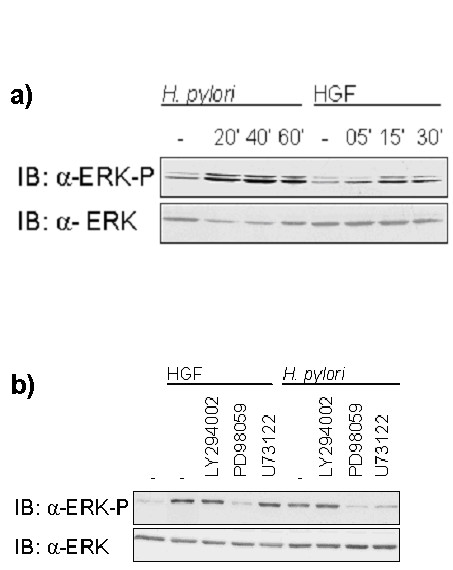
**Differential regulation of ERK1/2 activity in response to stimulation with HGF and *H. pylori *infection**. a) MDCK cells were infected with *H. pylori *or stimulated with HGF as indicated, or left untreated. Total cell lysates were subjected to SDS-PAGE and immunoblotted. The basal level of ERK was used as load control. b) MDCK epithelial cells were pretreated with pharmacological inhibitors of PI3K (LY294002), MEK (PD98059) or PLCγ1 (U73122) and infected with *H. pylori *or activated by HGF. The basal level of ERK was used as load control. Western blot analysis shows that inhibition of PI3K has no effect on ERK1/2 activation. Treatment of the cells with MEK inhibitor blocks ERK1/2 activation by *H. pylori *and HGF. Inhibition of PLCγ1 blocks ERK1/2 activation in *H. pylori *transfected cells, but not in HGF activated cells.

MDCK cells were used as a model system for the epithelial cells of the human gastric mucosa. The cells were stimulated with HGF or infected with *H. pylori *and the effect on ERK1/2 phosphorylation was evaluated via Western Blot analysis after cell lysis using anti-ERK1/2 and phospho-specific ERK1/2 antibodies. As shown in Figure 7a, ERK1/2 was activated after stimulation of the cells with HGF, as well as after infection with *H. pylori*, as demonstrated by the Western Blot analysis.

We then repeated the above experiments with different specific inhibitors to inhibit MEK, PI3K and PLCγ1. When treating the cells with the pharmacological inhibitor PD98059, which specifically inhibits MEK, our experimental results clearly indicated that ERK1/2 phophorylation was effectively reduced in the case of HGF stimulation as well as after infection with *H. pylori *(Figure 7b). This corresponds to what we expect since MEK is the only upstream effector of ERK1/2 in our model.

Inhibition of PI3K using the specific inhibitor LY294002 had no effect on ERK1/2 phosphorylation for both stimuli, again in agreement with the model prediction.

For the inhibition of PLCγ1 we used the specific inhibitor U73122. The Western Blot analysis could show that in the case of HGF stimulation, ERK1/2 activation was similar strong as in the untreated cells, but in the case of *H. pylori *infection ERK1/2 phophorylation was strongly reduced (Figure 7b). This result confirms the prediction of the *in silico *knockout of PLCγ1 in the logical model, which predicted no effect in the case of HGF simulation and a suppressive effect in the case of *H. pylori *infection (Figures 5 and 6).

## Conclusion

A logical model of HGF- and *H. pylori *induced c-Met signal transduction was presented. We used the formalism of logical interaction hypergraphs for network representation and analysis and it turned out that this qualitative approach is suitable for analysing a number of important aspects in this signalling network.

In a case study we could demonstrate the capability of the model to identify targets for ERK1/2 deactivation using the formalism of minimal intervention sets. Three network species (PI3K, MEK and PLCγ1) were chosen for *in silico *knockout. The effects on ERK1/2 activity predicted from the model for both HGF and *H. pylori *stimulus could all be confirmed by experimental results in MDCK cell culture, demonstrating the suitability of our logical model to generate *in silico *predictions for the effect of certain knockouts of species. This approach can also be used for the *in silico *identification of new targets against pathogen infection, as demonstrated here by the identification of PLCγ1 as a target for ERK1/2 deactivation. Inhibition of PLCγ1 prevents *H. pylori *induced cell scattering, providing a possible intervention strategy against invasive gastric cancer.

As a major finding, our work shows that PLCγ1 represents a key factor in *H. pylori*-induced ERK activation. Further, the identification of PLCγ1 as a target for inactivation of ERK could also have therapeutic implications. Inhibition of ERK1/2, which is critically important for the induction of cell scattering in *H. pylori*-infected epithelial cells, could represent a target for the treatment of invasive stomach cancers caused by *H. pylori *infection.

In the light of our observation, that the activation of ERK by growth factor induced c-Met signalling is not influenced by a PLCγ1 inhibition, the use of specific pharmaceutical inhibitors for PLCγ1 might thus represent a promising therapeutic strategy to prevent the pathogenic effects in *H. pylori *infection. Furthermore, *H. pylori *eradication therapy however is complicated by increasing global antibiotic resistance in the pathogen. Understanding of important epithelial signal transduction pathways (e.g. c-Met) modulated by *H. pylori *could delineate potential chemopreventative agents to target oncogenic pathways. Therefore, our study discovered PLCγ1 as a potential novel therapeutic target that has the advantage that the microorganism can hardly develop resistances.

This work represents one of the first approaches in the direction of host-pathogen systems biology and is a first step to decipher signalling changes brought about by pathogenic bacteria. The need for the identification of new targets in treating pathogen infection in light of the growing emergence of resistance against antibiotics is a formidable task for the discovery of novel anti-infectives. In this context host-pathogen systems biology can provide highly relevant clues, not only for the elucidation how the pathogen captures the host signalling mechanisms, but also for the identification of novel targets for therapeutic interventions.

In our future work, the presented logical network will be systematically extended. This includes in particular the incorporation of relevant processes related to the cytoskeleton, tight junctions and adherence junctions. The three output nodes α-catenin/β-catenin/E-cadherin, Talin and Calpain2 represent starting points for this future work.

## Methods

### Model generation, data collection and meta-analysis

Literature mining for the assembling of the biochemical data for the construction of the network model was done using databases for protein-protein interaction data and by manually searching the published literature in the relevant fields through Pubmed. We used two comprehensive, manually literature-curated databases on human protein-protein interaction, the Human Protein Reference Database (HPRD.org) [77] and IntAct [78]. For a query usually the protein name or the gene name respectively was used and the obtained hits were all evaluated manually by checking the primary literature.

Published data were evaluated carefully and only approved results, which are not contradicted by recently published data, were incorporated into the model. Only results obtained from epithelial cell cultures were considered for the model. This meta-analysis of the utilized experimental data included evaluation of all the primary data and published figures. Only data that were consistent and in agreement with the current knowledge were taken.

### Building and analysing the network model using CellNetAnalyzer

The logical network model (Table 1 and Figure 1) was implemented and analyzed with *CellNetAnalyzer *[13,14,79]. The network diagram was drawn using the CellDesigner Software (Version 3.5, The Systems Biology Institute, Tokyo, Japan) and then exported as an image file to *CellNetAnalyzer*.

### Experimental part: materials and methods

#### Cell stimulation, and H. pylori infection

MDCK (Madin-Darby Canine Kidney) cells were grown in RPMI 1640 medium containing 4 mM glutamin (Invitrogen), 100 U ml^-1 ^penicillin, 100 μg ml^-1 ^streptomycin, and 10% FCS (Invitrogen) in a humidified 5% CO_2 _atmosphere at 37°C. The cells were seeded in tissue culture plates for 48 h before infection. 16 h before infection, the medium was replaced by fresh RPMI 1640 without serum.

*H. pylori *wild-type strain P1 [6] was cultured on agar plates containing 10% horse serum under microaerophilic conditions at 37°C for 48 h. For the infection, bacteria were harvested in PBS, pH 7.4, and added to the host cells at a multiplicity of infection of 100.

The cells were infected with *H. pylori*, or were treated with 50 ng/ml HGF (Calbiochem). Pharmacological inhibitors for the inhibition of MEK (PD98059, 50 μM, Calbiochem), PLCγ1 (U73122, 5 μM, Calbiochem), and PI3K (LY294002, 25 μM, Calbiochem) were added to the cells 30 min before stimulation with HGF, or infection with *H. pylori*.

#### Cell lysis and Western Blots

For Western Blot analysis, MDCK cells were harvested at different time points after infection, and stimulation with HGF respectively, in lysis buffer (50 mM Tris-HCl, pH 7.5, 5 mM EDTA, 100 mM NaCl, 1% Triton X-100, and 10% glycerol) containing 2 mM Na_3_VO_4_, 1 mM PMSF, 1 mg/ml aprotinin, and 1 mg/ml pepstatin. Total cell lysates were subjected to SDS-PAGE and immunoblotting. Immunoblots were developed using enhanced chemiluminescence (ECL, Amersham Biosciences). Antibodies used in this work were anti-ERK 2 (K-23) (Santa Cruz Biotechnology), and phospho-p44/p42 MAPK (Thr202/Tyr204) antibody (Cell Signaling). The secondary antibody used was HRP conjugated anti-rabbit (Dianova).

## Abbreviations

AKT1 Protein kinase B

ATF2 activating transcription factor 2

Ca^2+ ^Calcium-Ions

CagA cytotoxine-associated gene A

c-Cbl Cas-Br-M (murine) ecotropic retroviral transforming sequence

c-JUN v-jun sarcoma virus 17 oncogene homolog

c-Met met proto-oncogene (hepatocyte growth factor receptor)

c-Met/HGF Complex

c-Myc avian myelocytomatosis virus oncogene cellular homolog

CNA CellNetAnalyzer

Crk/CrkL v-crk sarcoma virus CT10 oncogene homolog

CSK c-src tyrosine kinase

c-SRC v-src sarcoma (Schmidt-Ruppin A-2) viral oncogene homolog

DAG Diacylglycerol

DOCK180 dedicator of cytokinesis 1

ELK1 member of ETS oncogene family

ERK1/2 extracellular signal-regulated kinase 1/2

ETS E26-AMV virus oncogene cellular homolog

FAK focal adhesion kinase

FYN FYN oncogene related to SRC, FGR, YES

Gab1 GRB2-associated binding protein 1

Grb2 growth factor receptor-bound protein 2

*H. pylori *Helicobacter pylori

HGF Hepatocyte growth factor

IκBα Inhibitor of κBα

IKK IκB kinase

ILK Integrin-linked kinase

IP3 Inositol-1,4,5-triphosphat

IQGAP-1 IQ motif containing GTPase activating protein 1

JNK c-Jun kinase

MEK mitogen-activated protein kinase kinase 1

MKK4 Mitogen-activated protein kinase kinase 4

NF-κB Nuclear factor κB

PAK1 p21-activated kinase 1

PDK1 3-Phosphoinositide-dependent protein kinase-1

PI3K phosphoinositide-3-kinase, regulatory subunit 1 (p85 alpha)

PIP2 Phosphatidylinositol-4,5-bisphosphat

PIP3 Phosphatidylinositol (3,4,5) trisphosphate

PIPKIγ661 type I phosphatidylinositol phosphate kinase isoform-γ661

PKCalpha protein kinase C alpha

PLCγ1 phospholipase C gamma 1

PTEN Phosphatase and tensin homolog

RAC1 ras-related C3 botulinum toxin substrate

Raf1 v-raf-1 murine leukemia viral oncogene homolog 1

RasGAP Ras GTPase activating protein

Shc1 SHC (Src homology 2 domain containing) transforming protein 1

SHP2 SH2 containing protein tyrosine phosphatase 2

SOS1 son of sevenless homolog 1

STAT3 signal transducer and activator of transcription 3

## Authors' contributions

RF constructed and analysed the network model and was in charge of writing the manuscript. MM and TK constructed the network model, collected the data and contributed to the manuscript. NW carried out the experimental contributions. SK helped in the construction and the analysis of the logical model and wrote theoretical parts of the manuscript. EDG contributed with suggestions in model analysis. MN conceived and coordinated the study, participated in its design, and supervised the writing process of the manuscript. All authors have read and approved the final version of the manuscript.
